# Effects of a Soluble Epoxide Hydrolase Inhibitor on Lipopolysaccharide-Induced Acute Lung Injury in Mice

**DOI:** 10.1371/journal.pone.0160359

**Published:** 2016-08-04

**Authors:** Wei Tao, Ping-Song Li, Liu-Qing Yang, Yong-Bo Ma

**Affiliations:** 1 Department of Burn and Plastic Surgery, Subei People’s Hospital of Jiangsu province, Jiangsu, People’s Republic of China; 2 Department of Anesthesiology, Subei People’s Hospital of Jiangsu province, Jiangsu, People’s Republic of China; 3 Department of Outpatient, Subei People’s Hospital of Jiangsu province, Jiangsu, People’s Republic of China; Indian Institute of Integrative Medicine CSIR, INDIA

## Abstract

**Objectives:**

Inflammation plays a key role in the pathogenesis of acute lung injury (ALI). Soluble epoxide hydrolase (sEH) is suggested as a vital pharmacologic target for inflammation. In this study, we determined whether a sEH inhibitor, AUDA, exerts lung protection in lipopolysaccharide (LPS)-induced ALI in mice.

**Methods:**

Male BALB/c mice were randomized to receive AUDA or vehicle intraperitoneal injection 4 h after LPS or phosphate buffered saline (PBS) intratracheal instillation. Samples were harvested 24 h post LPS or PBS administration.

**Results:**

AUDA administration decreased the pulmonary levels of monocyte chemoattractant protein (MCP)-1 and tumor necrosis factor (TNF)-α. Improvement of oxygenation and lung edema were observed in AUDA treated group. AUDA significantly inhibited sEH activity, and elevated epoxyeicosatrienoic acids (EETs) levels in lung tissues. Moreover, LPS induced the activation of nuclear factor (NF)-κB was markedly dampened in AUDA treated group.

**Conclusion:**

Administration of AUDA after the onset of LPS-induced ALI increased pulmonary levels of EETs, and ameliorated lung injury. sEH is a potential pharmacologic target for ALI.

## Introduction

Inflammation plays a key role in the pathogenesis of acute lung injury (ALI) and its severe form acute respiratory distress syndrome (ARDS) [1]. Direct or indirect injury to the lung such as pneumonia, trauma, or sepsis, can cause pulmonary inflammation [1]. It is suggested that dysregulated and excessive inflammatory response may trigger a large number of activated inflammatory cells infiltrate in the lung and release cytokines, proteinases, and reactive oxygen species (ROS) [1, 2]. These factors enhance alveolar-capillary permeability, leading to exudation of the blood component into the alveolar space and pulmonary edema [3], resulting in refractory hypoxemia and high mortality which are the hallmark feature of ALI/ARDS [1]. Thus, novel strategies for the treatment of ALI/ARDS are desirable.

Soluble epoxide hydrolase (sEH) is a bifunctional homodimeric enzyme with both C-terminus epoxide hydrolase and N-terminus phosphatase activity [4]. The epoxide hydrolase motif of sEH transforms epoxyeicosatrienoic acids (EETs), an endogenous anti-inflammatory mediator, to the corresponding dihydroxyeicosatrienoic acids (DHETs) [4]. It has been reported that 14, 15-EET enhanced biosynthesis of 15-epi-lipoxin A (4) an anti-inflammatory and pro-resolving mediator [5]. Inhibition of sEH may lead to elevated levels of EETs, which in turn could elicit anti-inflammatory effects [6]. Meanwhile, in addition to its sEH inhibitory properties, inhibitors of sEH have now been proved to possess other anti-inflammatory activities. Inhibition of sEH attenuates bleomycin-induced mouse pulmonary fibrosis by reduce pulmonary inflammation and collagen deposition [7]. Roche and coworkers reported that sEH inhibitors (t-AUCB) increased the expression of IκB, the inhibitor of NF-κB, in an animal model of diabetic nephropathy [8]. However, it remains unknown whether sEH inhibitors have any protective effects on ALI. The present study was conducted to test the hypothesis that AUDA (12-(3-adamantan-1-yl-ureido)-dodecanoic acid), a sEH inhibitor, attenuates LPS-induced ALI. We also investigate its potential mechanism.

## Methods

### Experimental Protocol

Healthy male BALB/c mice (17–20 g, 6 to 8-week-old) were purchased from Yangzhou University. The mice were housed separately and maintained on a 12 h light/ 12 h dark schedule in a specific pathogen-free environment and received standard laboratory rodent chow *ad libitum*. To minimize the suffering of mice during experiments, all operations were performed under anesthesia. The mice were randomly divided into various groups: control group, vehicle group, and AUDA group. Animals were anesthetized by intraperitoneal injection of carbrital (20 mg/kg). Then, ALI was induced by 50 μl of lipopolysaccharide (LPS, Sigma, St. Louis, MO, USA.) intratracheal instillation as described previously [9]. LPS was dissolved in sterilized phosphate buffered saline (PBS) at 10 mg/ml before use. Equivalent PBS was administrated for control group. Four hours after LPS or PBS challenge, AUDA (10 mg/kg) or vehicle (2-hydroxypropyl)-bcyclodextrin (Sigma, St Louis, MO, USA) (200 μl/mouse) intraperitoneal injection was performed. The dose of AUDA selected in the present study was based on a previously published article [10]. Before use, AUDA was dissolved in (2-hydroxypropyl)-bcyclodextrin at 5 mg/ml [10]. AUDA was synthesized as described previously [11]. To investigate the effect of AUDA on pulmonary microvascular albumin-permeability, we performed the Evan’s blue (EB) (50 μg/g) dye technique 30 min prior to sacrifice as previously described [12]. EB content in lung tissues was measured spectrophotometrically and calculated (μg EB/g lung/min) after sacrifice. Twenty-four hours after LPS or PBS challenge, mice in each group were sacrificed by cervical dislocation and exsanguinated by cutting the vena cava inferior for tissue and blood sampling. Blood sampling was examined immediately by a blood gas analyzer (FM-7700 Blood Gas Electrolyte Analyzer, Anhui, China). We investigated oxygenation by calculated the partial pressure of arterial oxygen (PaO_2_)/fraction of inspiration O_2_ (FiO_2_) ratio (at twenty-four h after LPS or saline challenge). As the animals were housed in a room with normal atmosphere, we set the FiO_2_ as 0.21. Lung tissue sampling was homogenized in PBS on ice to make the 10% pulmonary homogenate, and stored at −70°C for further analysis. All experiments were performed according to the guidelines for the care and use of animals as established by the Animal Ethics Committee of Yangzhou University. The animals used for our study protocol was approved by the Animal Ethics Committee of Yangzhou University.

### Histological Examination

For immediate fixation, the lung specimen was submerged in 10% formalin, then, dehydrated in alcohol, and later embedded in paraffin. The fixed tissue block was then sectioned at a thickness of 5 μm, floated on warm water, and transferred to glass slides. The lung section was stained with H&E and examined with light microscopy. The Murakami technique [13], which is based on the following histologic features: edema, congestion, infiltration of inflammatory cells, and hemorrhage, was employed to grade the degree of lung injury by two blinded examiners. Each feature was graded as: 0, absent and appears normal; 1, light; 2, moderate; 3, strong; 4, intense. A total score was calculated for each animal.

### Myeloperoxidase Activity Analysis

Myeloperoxidase (MPO) activities, the indicator of neutrophil accumulation, in lung homogenates were measured according to the manufacturers’ instructions (R&D Systems Inc., Minneapolis, MN, USA) and calculated from the absorbance changes resulting from decomposition of hydrogen peroxide in the presence of odianisidine.

### Measurement of Inflammatory Mediator Levels

The levels of monocyte chemoattractant protein (MCP)-1 and tumor necrosis factor (TNF)-α in pulmonary tissue were determined by using ELISA kits (R&D Systems Inc, Minneapolis, MN, USA) according to the manufacturers’ manual.

### NF-κB Binding Assay

The DNA-binding activity of NF-κB p65 was determined using an ELISA-based NF-κB p65 transcription factor assay kit (Active Motif North America) according to the manufacturers’ manual.

### EETs Measurement and sEH Activity Analysis

The levels of EETs and sEH activity in lung homogenates were measured using a 14, 15-EET/DHET ELISA kit (Detroit R&D, Michigan, USA) according to the manufacturers’ manual. An indirect method was used to detect the levels of EETs. Briefly, using the 14, 15-DHET ELISA kit to measure DHET (value 1), which includes DHET converted from EET. At the same time, measure the DHET level without hydrolysis of EET in the same sample (value 2). The EET level in the sample could be calculated by subtract value 2 from value 1. The sEH activity was also measured by an indirect method as follows. In brief, cytosol was extracted from frozen lung tissue with a cytosol extraction kit (BioVision, CA, USA). 50μl cytosol was added to 100μl of 1X sample dilution buffer incubated for 1 h with shaking at room temperature. 14, 15-EET was added to a final concentration of 1uM and incubation at 37°C for 30 min. Then, 100μl of media was transferred to the 14, 15-DHET ELISA plate. 14, 15-DHETs were quantified by use of a 4000 QTRAP tandem mass spectrometer at 450nm (Applied Biosciences).

### Statistical Analyses

All data were analyzed with SPSS 17.0 (IBM, Armonk, USA). Data were presented as mean±SEM. Statistical significance between different groups was carried out using one-way analysis of variance (ANOVA) followed by the Student-Newman-Keuls method. The histopathologic scores were carried out using Kruskal-Wallis one-way analysis of variance on ranks and the Student-Newman-Keuls method. P value less than 0.05 was considered to be statistically significant.

## Results

### Effects of AUDA on ALI

**Fig 1:**
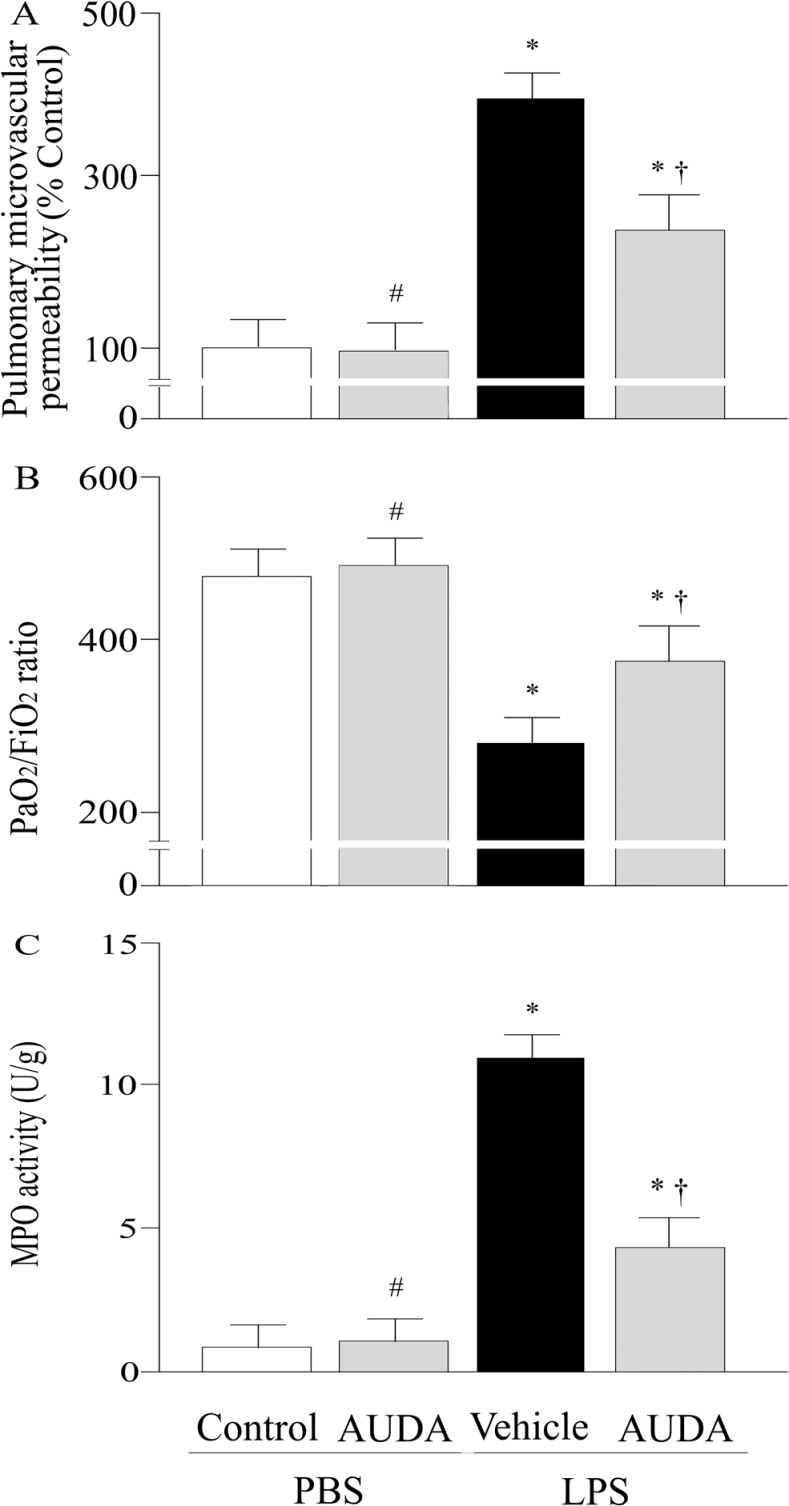
Alterations of pulmonary microvascular albumin-permeability (a), or the partial pressure of arterial oxygen (PaO_2_)/fraction of inspired O_2_ (FiO_2_) ratio (b), myeloperoxidase (MPO) activity (c) of control or lung injury animals treated with vehicle or AUDA 24 h after lipopolysaccharide (LPS) or phosphate buffered saline (PBS) challenged. Data are expressed as the mean±SEM (n = 6~8) and compared by one-way analysis of variance and the Student-Newman-Keuls method. * P<0.05 when compared with control group; # P>0.05 when compared with control group; † P<0.05 when compared with vehicle group.

**Fig 2:**
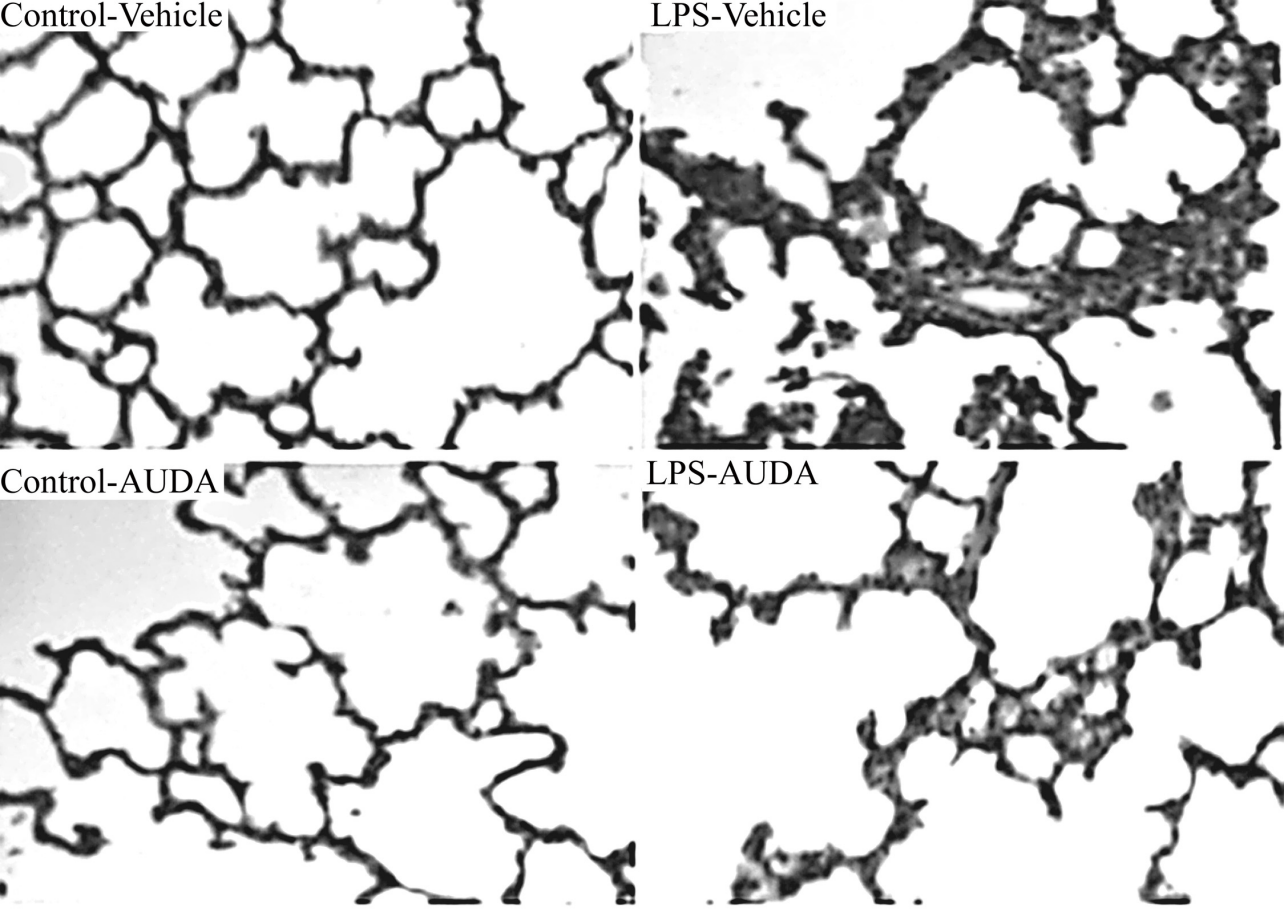
Morphologic alterations of the lungs were determined by photomicrography (400× magnification). Infiltration of neutrophils and pulmonary septal widening were presented in lipopolysaccharide (LPS) +vehicle group 24 h after LPS challenged. These changes were improved in AUDA treated group.

**Fig 3:**
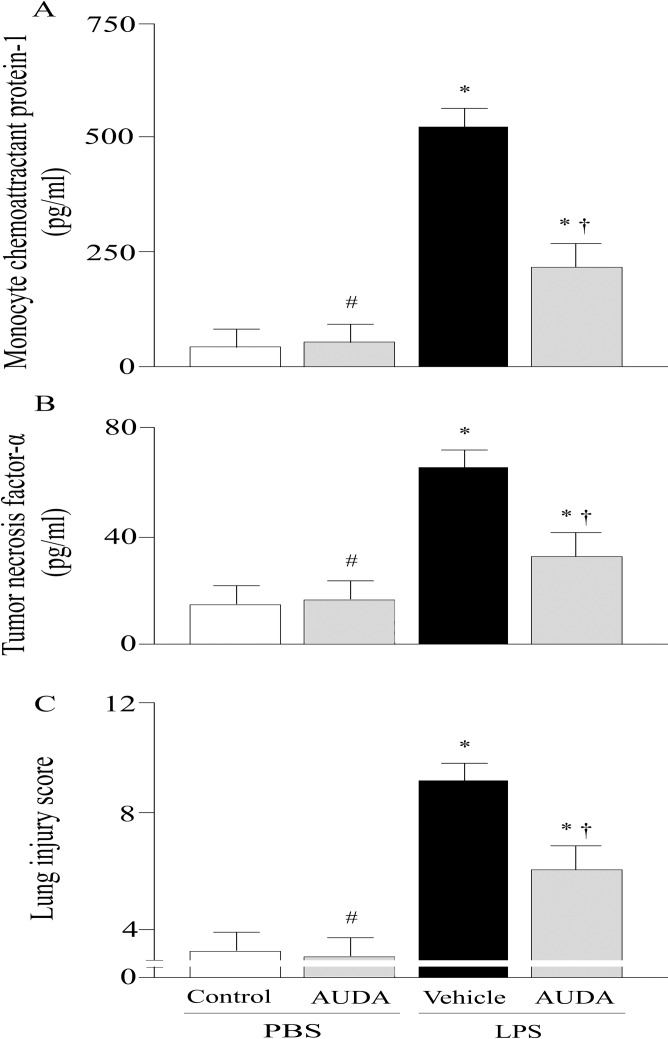
Alterations of monocyte chemoattractant protein (MCP)-1 levels (a), or tumor necrosis factor (TNF)-α levels (b), and histopathologic scoring (c) in control or lipopolysaccharide (LPS)-challenged animals treated with vehicle or AUDA. Data are expressed as the mean±SEM (n = 6~8) and compared by one-way analysis of variance and the Student-Newman-Keuls method or Kruskal-Wallis one-way analysis of variance on ranks and the Student-Newman-Keuls method. * P<0.05 when compared with control group; # P>0.05 when compared with control group; † P<0.05 when compared with vehicle group. PBS, phosphate buffered saline.

Pulmonary microvascular albumin-permeability that measured by the Evan’s blue dye technique, and PaO_2_/ FiO_2_ ratio were significantly improved by AUDA treatment at twenty-four h after LPS challenge (Fig 1A and 1B). LPS challenge dramatically increased pulmonary MPO activities, while this effect was remarkably attenuated by AUDA administration (P<0.05; Fig 1C). The lung injury score was significantly improved in AUDA-treated groups compared with vehicle group (P<0.05; Figs 2 and 3C).

### Effects of AUDA on Pulmonary Inflammatory Mediators Levels

The effects of AUDA on MCP-1 and TNF-α level were measured by using ELISA. Compared to control group, the levels of MCP-1 and TNF-α were increased by 12.8-fold (P<0.05; Fig 3A) and 5.3-fold (P<0.05; Fig 3B), respectively, in vehicle group. The pulmonary MCP-1 and TNF-α levels were markedly reduced in AUDA treated group (P<0.05; Fig 3A and 3B).

### Effects of AUDA on EETs and sEH Activities

**Fig 4:**
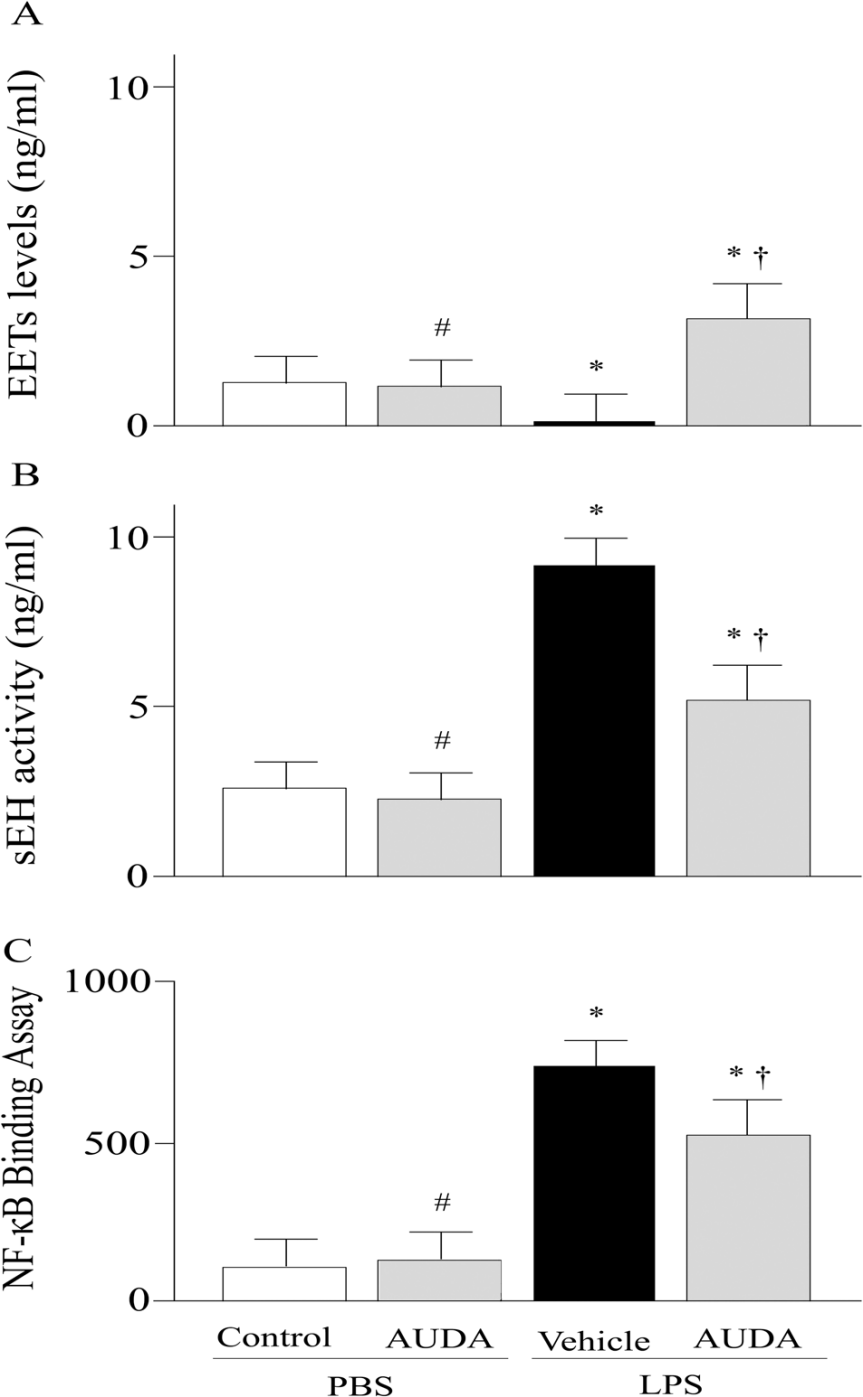
Alterations of epoxyeicosatrienoic acids (EETs) (a), or soluble epoxide hydrolase (sEH) activity (b), and nuclear factor (NF)-κB activity (c) in control or lipopolysaccharide (LPS)-challenged animals treated with vehicle or AUDA. Data are expressed as the mean±SEM (n = 6~8) and compared by one-way analysis of variance and the Student-Newman-Keuls method. * P<0.05 when compared with control group; # P>0.05 when compared with control group; † P<0.05 when compared with vehicle group. PBS, phosphate buffered saline.

LPS markedly reduced the EETs levels (Fig 4A). The AUDA treated group had significantly increased EETs levels compared with the vehicle group (Fig 4A). The activity of sEH was much greater in vehicle group compared with control group (P<0.05; Fig 4B); the LPS-induced elevation of sEH activities was significantly reduced in AUDA treated group. In PBS challenged group, AUDA reduced sEH activity but showed no statistically significant effects (Fig 4B).

### Effects of AUDA on NF-κB Binding Activity

LPS significantly induced NF-κB DNA-binding activity in vehicle group compared with control group (P<0.05; Fig 4C); the NF-κB DNA-binding activity was remarkably reduced in AUDA treated group (P<0.05; Fig 4C).

## Discussion

LPS triggered pulmonary edema, deteriorated oxygenation, and infiltration of inflammatory mediators are present in vehicle-treated group. These injuries are associated with decreased levels of EETs in the pulmonary tissues. Treatment of the AUDA markedly increases EETs levels and offers a protective role against LPS-induced ALI in mice. Furthermore, our result suggests that the beneficial effect of AUDA, in addition to inhibit sEH, may partly associate with inhibition of NF-κB pathway.

sEH is suggested as a vital pharmacologic target for inflammation [14]. EETs are the substrate of sEH [15]. The anti-inflammatory feature of EETs is well defined in a number of studies [16–18]. Compared with vehicle-treated group, AUDA treatment markedly increased the level of EETs. And improved lung injury was detected in AUDA treated group that with significantly elevated EETs. Thus, it seems that EETs plays an important effect in the pathophysiological functions of lungs. It has been reported that EETs can inhibit inflammatory cells activation and adhesion to the vascular wall, and the anti-inflammatory effect of EETs may be mediated via inhibition of NF-κB pathway [17]. Evidence has shown that endotoxin-induced activation of NF-κB signaling pathway, chemokine and cytokine expression, and neutrophil infiltration was significantly dampened in sEH gene deficiency mice [18]. sEH inhibition can reduce bleomycin-induced pulmonary inflammatory cells infiltration and interleukin (IL)-1β and IL-6 levels in the serum [7].

Considerable evidence has indicated that NF-κB activation is a logical therapeutic target for ALI [19, 20]. In the present study, the DNA-binding activity of NF-κB p65, an indicator of NF-κB activation, was significantly inhibited in AUDA treated group compared with vehicle. This result suggested a potential effect of AUDA on inhibition of NF-κB activation. However, its potential mechanisms are not well defined. Roche and coworkers reported that sEH inhibitors (t-AUCB) increased the expression of IκB, the inhibitor of NF-κB, in an animal model of diabetic nephropathy [8]. Normally, NF-κB is sequestered in the cytoplasm and binding with its inhibitor IκB. When cells are stimulated by LPS, the IκB is phosphorylated, result in NF-κB freed from association with it, then, NF-κB moves to the nucleus and binds to specific genes sequences in the promoter/enhancer regions [21, 22]. Inhibition of NF-κB pathway prevents multiple proinflammatory gene expression [23]. In our study, the inhibition of NF-κB activation was accompanied with reduced levels of TNF-α and MCP-1. And the reduced proinflammatory cytokines levels may contribute to the dampened pulmonary inflammation and injury. Thus, the protective effect of AUDA on LPS-induced ALI may, partly, mediate through inhibition of NF-κB.

## Conclusions

Administration of AUDA after the onset of LPS-induced ALI increased pulmonary levels of EETs, ameliorated lung injury. sEH is a potential pharmacologic target for ALI.

## References

[1] Ware LB, Matthay MA (2000) The acute respiratory distress syndrome. N Engl J Med 342: 1334–1349. 10793167 10.1056/NEJM200005043421806

[2] Tao W, Miao QB, Zhu YB, Shu YS (2012) Inhaled neutrophil elastase inhibitor reduces oleic acid-induced acute lung injury in rats. Pulm Pharmacol Ther 25: 99–103. 10.1016/j.pupt.2011.12.006 22210005

[3] Zemans RL, Colgan SP, Downey GP (2009) Transepithelial migration of neutrophils: mechanisms and implications for acute lung injury. Am J Respir Cell Mol Biol 40: 519–535. 10.1165/rcmb.2008-0348TR 18978300 PMC2677434

[4] Newman JW, Morisseau C, Hammock BD (2005) Epoxide hydrolases: their roles and interactions with lipid metabolism. Prog Lipid Res 44: 1–51. 15748653 10.1016/j.plipres.2004.10.001

[5] Planagumà A, Pfeffer MA, Rubin G, Croze R, Uddin M, Serhan CN, et al (2010) Lovastatin decreases acute mucosal inflammation via 15-epi-lipoxin A4. Mucosal Immunol 3: 270–279. 10.1038/mi.2009.141 20130564 PMC3260795

[6] Larsen BT, Gutterman DD, Hatoum OA (2006) Emerging role of epoxyeicosatrienoic acids in coronary vascular function. Eur J Clin Invest 36: 293–300. 16634832 10.1111/j.1365-2362.2006.01634.x

[7] Zhou Y, Sun GY, Liu T, Duan JX, Zhou HF, Lee KS, et al (2016) Soluble epoxide hydrolase inhibitor 1-trifluoromethoxyphenyl-3- (1-propionylpiperidin-4-yl) urea attenuates bleomycin-induced pulmonary fibrosis in mice. Cell Tissue Res 363(2): 399–409. 10.1007/s00441-015-2262-0 26310139 PMC4738109

[8] Roche C, Guerrot D, Harouki N, Duflot T, Besnier M, Rémy-Jouet I, et al (2015) Impact of soluble epoxide hydrolase inhibition on early kidney damage in hyperglycemic overweight mice. Prostaglandins Other Lipid Mediat 120: 148–54. 10.1016/j.prostaglandins.2015.04.011 26022136 PMC4575616

[9] Lin WC, Lin CF, Chen CL, Chen CW, Lin YS (2011) Inhibition of neutrophil apoptosis via sphingolipid signaling in acute lung injury. J Pharmacol Exp Ther 339: 45–53. 10.1124/jpet.111.181560 21724966

[10] Jung O, Brandes RP, Kim IH, Schweda F, Schmidt R, Hammock BD, et al (2005) Soluble epoxide hydrolase is a main effector of angiotensin II-induced hypertension. Hypertension 45: 759–765. 15699457 10.1161/01.HYP.0000153792.29478.1d

[11] Morisseau C, Goodrow MH, Newman JW, Wheelock CE, Dowdy DL, Hammock BD (2002) Structural refinement of inhibitors of urea-based soluble epoxide hydrolases. Biochem Pharmacol 63: 1599–1608. 12007563 10.1016/s0006-2952(02)00952-8

[12] Wang L, Taneja R, Razavi HM, Law C, Gillis C, Mehta S (2012) Specific role of neutrophil inducible nitric oxide synthase in murine sepsis-induced lung injury in vivo. Shock 37: 539–547. 10.1097/SHK.0b013e31824dcb5a 22392143

[13] Muller DN, Schmidt C, Barbosa-Sicard E, Wellner M, Gross V, Hercule H, et al (2007) Mouse Cyp4a isoforms: enzymatic properties, gender- and strain-specific expression, and role in renal 20-hydroxyeicosatetraenoic acid formation. Biochem J 403: 109–118. 17112342 10.1042/BJ20061328 PMC1828894

[14] Schmelzer KR, Kubala L, Newman JW, Kim IH, Eiserich JP, Hammock BD (2005) Soluble epoxide hydrolase is a therapeutic target for acute inflammation. Proc Natl Acad Sci U S A 102: 9772–7. 15994227 10.1073/pnas.0503279102 PMC1168955

[15] Decker M, Arand M, Cronin A (2009) Mammalian epoxide hydrolases in xenobiotic metabolism and signalling. Arch Toxicol 83: 297–318. 10.1007/s00204-009-0416-0 19340413

[16] Morisseau C, Hammock BD (2013) Impact of Soluble Epoxide Hydrolase and Epoxyeicosanoids on Human Health. Annu Rev Pharmacol Toxicol 53: 37–58. 10.1146/annurev-pharmtox-011112-140244 23020295 PMC3578707

[17] Node K, Huo Y, Ruan X, Yang B, Spiecker M, Ley K, et al (1999) Anti-inflammatory properties of cytochrome P450 epoxygenase-derived eicosanoids. Science 285: 1276–9. 10455056 10.1126/science.285.5431.1276 PMC2720027

[18] Deng Y, Edin ML, Theken KN, Schuck RN, Flake GP, Kannon MA, et al (2011) Endothelial CYP epoxygenase overexpression and soluble epoxide hydrolase disruption attenuate acute vascular inflammatory responses in mice. FASEB J 25:703–13. 10.1096/fj.10-171488 21059750 PMC3023387

[19] Fan J, Ye RD, Malik AB (2001) Transcriptional mechanisms of acute lung injury. Am J Physiol Lung Cell Mol Physiol 281: L1037–L1050. 11597894 10.1152/ajplung.2001.281.5.L1037

[20] Shu YS, Tao W, Miao QB, Zhu YB (2014) Improvement of ventilation-induced lung injury in a rodent model by inhibition of inhibitory κB kinase. J Trauma Acute Care Surg 76: 1417–1424. 10.1097/TA.0000000000000229 24854310

[21] Baeuerle PA, Baichwal VR (1997) NF-kappa B as a frequent target for immunosuppressive and anti-inflammatory molecules. Adv Immunol 65: 111–137. 9238509

[22] Blackwell TS, Christman JW (1997) The role of nuclear factorkappa B in cytokine gene regulation. Am J Respir Cell Mol Biol 17: 3–9. 9224203 10.1165/ajrcmb.17.1.f132

[23] Liu SF, Malik AB (2006) NF-kappa B activation as a pathological mechanism of septic shock and inflammation. Am J Physiol Lung Cell Mol Physiol 290: L622–L645. 16531564 10.1152/ajplung.00477.2005

